# Computer-assisted cognitive-behavioral therapy for adolescent depression in primary care clinics in Santiago, Chile (YPSA-M): study protocol for a randomized controlled trial

**DOI:** 10.1186/1745-6215-15-309

**Published:** 2014-08-05

**Authors:** Vania Martínez, Pablo Martínez, Paul A Vöhringer, Ricardo Araya, Graciela Rojas

**Affiliations:** Facultad de Medicina, Universidad de Chile, CEMERA, Profesor Zañartu 1030, Independencia, Santiago, Chile; Millennium Nucleus Psychological Intervention and Change in Depression, Av. Vicuña Mackenna 4860 Macul, Santiago, Chile; Clínica Psiquiátrica Universitaria, Universidad de Chile, Av. La Paz 1003, Recoleta, Santiago, Chile; Mood Disorders Program, Tufts Medical Center, Tufts University, Boston, USA; London School of Hygiene and Tropical Medicine, London, WC1E 7TH UK

**Keywords:** Adolescent depression, cognitive-behavioral therapy, computer-assisted therapy, depression treatment, primary care

## Abstract

**Background:**

Depression is a common and disabling condition. In Chile, assistance is guaranteed by law through a national program for depression in primary care services, and there is evidence of effective treatment for depressed women. However, there is a shortage of evidence-based treatments for depression in adolescents. The incorporation of technology to expand therapeutic options is becoming more common. This proposal aims to compare the efficacy of therapy that enhances traditional face-to-face cognitive-behavioral therapy (CBT) with a computer-based program versus usual care to treat depression in adolescents in primary care clinics in Santiago, Chile.

**Methods and design:**

This is a two-arm, single-blind, randomized controlled trial with a target enrollment of 216 depressed adolescents between 15 and 19 years of age, attending four primary care clinics in Santiago, Chile. In the active arm, depressed adolescents will receive eight sessions of computer-assisted CBT, led by trained psychologists on a weekly basis. In the control arm, depressed adolescents will receive treatment as usual from the primary care centers. Mean depression scores and indicators of dysfunctional thoughts, problem-solving strategies, and health-related quality of life will be measured at baseline and four and six months after randomization.

**Discussion:**

As far as we know, this is the first randomized controlled trial of a computer-assisted CBT intervention for depressed adolescents in a Latin American country.

**Trial registration:**

Clinical Trials: NCT01862913

## Background

Depression is a global public health concern, as it is a highly prevalent and recurrent condition that affects people of all ages and carries high individual, family, and social costs [1]. It is currently a leading cause of burden worldwide, measured in terms of disability-adjusted life years [2].

Key epidemiological studies have investigated the burden of depression in the Chilean general population [3, 4], and in recent years, research has reported a high prevalence of depression in adolescents between the ages of 12 and 18 [5].

To address the needs of the population, a National Depression Detection and Treatment Program in Chilean Primary Care was first introduced in 2001, consisting of a stepped care program that combines medical and psychosocial interventions, as well as improved detection, diagnosis, treatment, and follow-up of each case [6, 7]. In 2006, the treatment of depressive episodes, recurrent depressive disorder, and depressive episodes of bipolar disorder was strengthened with the introduction of the Regime of Explicit Health Guarantees (AUGE) [8]. This plan ensures access to opportune, quality, and financially protected depression treatment for all persons 15 years of age and older. In this plan, the primary care teams play a leading role in the management of depression. The AUGE Clinical Guidelines for Depression treatment in primary care include pharmacological treatment, medical controls, psychosocial interventions, and referral to secondary or tertiary psychiatric services, depending on the specificities of each case [9]. Additionally, it is important to note that, in Chile, there is evidence of effective psychosocial treatments based in primary care centers for depressed low-income women and for mothers affected by depression during the postnatal period [10, 11].

International studies have shown that adolescent depression is strongly linked to clinical and social morbidity [12, 13]. However, although several systematic reviews have demonstrated the efficacy of cognitive-behavioral therapy (CBT) interventions [14, 15], there has been little or no research into adolescent depression treatment options in Chile. One way to increase the appeal of CBT for adolescents could be to use computer-assisted therapy. This is especially important in primary care centers where CBT for adolescents with depression in routine clinical practice is limited [16]. Eells *et al.* define computer-assisted psychotherapy as a ‘psychosocial treatment in which a significant portion of the content is delivered with the use of computer technology or in which the computer technology assists the work of a therapist’ [17]. The incorporation of technology to expand therapeutic options is becoming more common. Computer-based or online programs have proven efficacy in the treatment of adults [18, 19], although the outcomes in adolescents remain unclear [20]. A pilot study has shown that adolescents could be motivated by the incorporation of technology into therapeutic sessions at their homes, guided by a professional who is not a CBT therapist, though the sample size of this study is quite low [21]. There has yet to be a randomized controlled trial to evaluate a procedure that enhances traditional face-to-face CBT with a computer program for adolescents in primary care centers.

Adding a computer-based component to face-to-face psychotherapy sessions may further favor the acceptance and incorporation of CBT therapy techniques. This combination would take advantage of the strengths of each approach: (1) the multi-media program could better hold the attention of the adolescents, while providing them with more stimuli and therefore an enhanced learning experience; and (2) the presence of a clinician permits the development of a therapeutic bond and allows for the tailoring of the program to the individual case. Furthermore, a systematic review and meta-analysis found that when computer-assisted therapy was combined with clinician support, the dropout rate was significantly lower than it was for programs without the presence of the therapist (28% versus 74%) [18]. For these reasons, we believe that the use of a computer-assisted CBT program, delivered in person by trained therapists, is a promising way to deliver these treatments among adolescents.

## Methods and design

This is a two-arm, single-blind (blinded only to outcome assessor), individually randomized controlled trial, which will compare the efficacy of computer-assisted CBT versus usual treatment for depression in adolescents in primary care clinics in Santiago, Chile.

### Aims and hypotheses

#### General aim

To carry out a randomized controlled trial to compare the efficacy of a computer-assisted CBT intervention versus usual care to treat depression in adolescents in primary care clinics in Santiago, Chile.

#### Specific aims

1. To compare the level of depressive symptoms of adolescents suffering depression treated with computer-assisted CBT versus usual care in primary care clinics.
2. To compare the level of dysfunctional thoughts, strategies for solving problems, and health-related quality of life (HRQoL) of adolescents suffering depression treated with computer-assisted CBT versus usual care in primary care clinics.
3. To compare adolescents’ adherence to computer-assisted CBT versus usual care in primary care clinics.
4. To compare adolescents’ satisfaction with computer-assisted CBT versus usual care in primary care clinics.

#### Hypotheses

1. Adolescents receiving the intervention will achieve lower scores (difference in mean of at least 0.4 standard deviations) in the depression questionnaire than those receiving the usual care, four months after randomization.
2. Adolescents receiving the intervention would have fewer dysfunctional thoughts than those receiving the usual care, four months after randomization.
3. Adolescents receiving the intervention would be better at solving problems than those receiving the usual care, four months after randomization.
4. Adolescents receiving the intervention would have better HRQoL than those receiving the usual care, four months after randomization.
5. Symptomatic improvements achieved at four months after randomization will be maintained at six months after randomization.

### Setting and population

Adolescents between 15 and 19 years of age attending four primary care clinics located in Puente Alto, a low-income municipality of Santiago, Chile, with a large adolescent population.

### Inclusion and exclusion criteria

Depressed adolescents aged between 15 and 19 years of age will be invited to participate. Informed consent will be obtained from each participant over 18 years of age, and from the parent or guardian of under-age participants. In the case of the latter, informed assent from the participants will also be required to continue with the study. Depression will be assessed with the Beck Depression Inventory (BDI) [22]. Adolescents scoring ten or more points on the BDI will be invited to a semi-structured diagnostic interview that includes the Kiddie-SADS-Present and Lifetime Version interview (K-SADS-PL) [23] to assess other inclusion criteria (diagnostic criteria for depressive disorders) and to ensure that they do not meet the exclusion criteria (current suicidal risk requiring in-patient care, current psychosis, alcohol or substance dependence, or low intellectual ability). Those taking antidepressants or receiving psychotherapy, and those with a history of bipolar illness, will also be excluded. The K-SADS-PL interview will be conducted with all potential participants by the principal investigator (VM), who is a child and adolescent psychiatrist, or a clinical psychologist, who have both been certified in conducting the interview.

### Training of primary care center professionals

Before the start of recruitment, professionals in the four participating primary care centers will receive a special training session from the principal investigator (VM) to assist with the correct identification, diagnosis, and treatment of patients with depression, according to the AUGE Clinical Guidelines for Depression. There will also be a refresher session 6 months after the start of recruitment.

### Intervention arm

The intervention arm will receive eight sessions of computer-assisted CBT plus usual medical treatment, as described in the AUGE Clinical Guidelines for Depression. Computer-assisted CBT sessions will be delivered on a weekly basis and assisted by trained psychologists in face-to-face meetings. The program is called ‘Yo pienso, siento y actúo mejor’ (YPSA-M); in English, ‘*I think, feel, and behave better*’. Topics covered in the program will include information on symptoms and causes of depression, treatment options, problem-solving techniques and other cognitive-orientated strategies to challenge negative thoughts. Copies of the computerized program are available upon written request to the principal investigator (VM).

Psychologists delivering the intervention will receive 12 hours of training and will be supervised by the study principal investigator (VM) once a month, and upon request.

Between sessions, members of the study team will contact the participants to remind them of upcoming appointments in the intervention arm.

### Control arm

The control arm will receive treatment as usual from the primary care clinics. The professionals in the primary care centers will be instructed to follow the AUGE Clinical Guidelines for Depression.

### Outcome measures

#### Primary outcome measures

Depressive symptoms will be assessed as a primary outcome using the BDI as a continuous variable. This inventory is designed to evaluate depressive symptoms in adults and adolescents from the age of 13. The BDI is a brief and well-established depression questionnaire translated into different languages and used widely throughout the world. It has shown good psychometric properties [22]. It has previously been used in primary care settings in Chile [24].

#### Secondary outcome measures

The Children’s Automatic Thoughts Scale is a self-reporting questionnaire to evaluate dysfunctional thoughts of children and adolescents. Internal consistency of the subscales is high, and the test-retest reliability is adequate [25]. The ‘personal failure’ subscale will be used as a secondary outcome measure. This subscale was chosen because it is most closely associated with depression. It has ten items. Each item is given a score of between 0 and 4 points and thus the total score varies between 0 and 40. Highest scores show a greater presence of negative automatic thoughts. In the validation study, this subscale discriminated well between the community sample and the sample of depressed individuals; the Cronbach’s alpha coefficient was 0.92. The test-retest correlation coefficient was 0.80 at one month and 0.74 at three months. The mean score in a non-clinical sample was 6.48 (standard deviation = 7.20) [25].

The Social Problem-Solving Inventory - Revised Short Form is a self-reporting questionnaire with 25 items. It consists of five subscales with five items each. Two of these subscales, ‘positive problem orientation’ and ‘negative problem orientation’, assess functional and dysfunctional cognitive and emotional orientations towards solving problems. The three remaining subscales, ‘rational problem solving’, ‘impulsivity-carelessness style’, and ‘avoidance style’, assess problem-solving skills and behavioral style. The total score of this scale varies between 0 and 20 points. Highest scores correspond to better social problem-solving abilities [26]. Calvete and Cardeñoso [27] used a Spanish version that had good psychometric properties and had been used in Chile. Cronbach’s alpha coefficient varied between 0.64 and 0.82 in the different subscales [27].

The KIDSCREEN-27 is a HRQoL questionnaire and consists of 27 items pertaining aspects of the child’s physical wellbeing, mood, autonomy, satisfaction with parental relationship and the atmosphere at home, perceived nature of the respondents’ relationships with other children or adolescents and perceptions of his or her cognitive capacity, learning and concentration, and his or her feelings about school. The KIDSCREEN items assess either the frequency of certain behaviors or feelings (never, seldom, sometimes, often, always) or, in fewer cases, the intensity of an attitude (not at all, slightly, moderately, very, extremely). The recall period is one week. For the scoring and analysis, negatively worded items will be recoded so that for all items, higher scores indicate a better HRQoL [28]. The KIDSCREEN has been validated in Chile [29].

### Other outcomes

The attendance of participants in both study arms to programmed appointments will be monitored, to evaluate treatment adherence. Additionally, satisfaction with treatment will be measured with a self-report questionnaire, six months after the baseline assessment.

### Sample size

We anticipate that the intervention, will produce a clinically meaningful improvement (remission) in 60% of adolescents and only 40% of those in usual care will reach this level of improvement in four months. A difference of 20% will be regarded as worth detecting and clinically meaningful. For a two-sided alpha coefficient of 5% and a statistical power of 80%, 97 adolescents will be needed in each group to detect this difference, or 108 subjects per group, allowing for 10% attrition. This total also yields an 80% power to detect a difference in mean of at least 0.4 standard deviations in the primary outcome measure.

### Recruitment

Adolescents eligible for the study will be identified by health professionals of the four primary care clinics, as well as by psychologists and counselors of nearby schools, who will be informed of the study, trained to identify potential cases of depression among their students, and instructed to refer any adolescent who seems to have depression symptoms to the primary care clinics for further evaluation and possible participation in the study, according to inclusion and exclusion criteria.

### Group assignment

Those adolescents who at baseline assessment meet the inclusion criteria and do not meet the exclusion criteria will be randomly assigned to the intervention arm or to the control arm. Blocked (size of four), stratified randomization will be used. Stratification will be implemented regarding sex and severity of depression (mild, moderate, and severe) according to BDI score. Randomization will be generated using web-based random allocation algorithms. Allocation concealment will be carried out by keeping treatment assignment in numbered sealed envelopes in a central place; the envelopes will be opened by individuals who do not participate in the recruitment process.

### Data collection

All participants will be assessed at baseline and at four and six months after randomization. The instruments that will be used are all self-report questionnaires, which will be completed on paper by the participants. Trained psychologists who are blind to the group assignments will be present to assist the adolescents if necessary.

### Data management

After the participants have completed the questionnaires, the data will entered into a secure platform, without identifying information (each participant will be assigned an ID number). The original copies of the instruments will be filed and stored, under lock and key, in the principal investigator’s office, along with the list linking the participants’ names and ID numbers. Only two research assistants, in charge of data entry, and the statistician will have access to the database.

### Data analysis

Data and presentation of the results will be in accordance with CONSORT guidelines for randomized clinical trials, with the primary comparative analysis being conducted on an intention-to-treat basis. Initially, we will conduct descriptive analysis to assess the balance between the two groups. The primary analysis will employ multivariable linear regression to investigate differences in mean symptom scores (primary outcome measure) between groups at four months after randomization, adjusting for baseline outcome variable if imbalances are identified. Sensitivity analysis making different assumptions will be conducted to investigate the potential effects of missing data. Similar analyses will be conducted for the secondary outcome measures.

### Monitoring

The primary care center staff will be told to report any type of adverse event (for example, hospitalization, pregnancy, suicide attempt) affecting the participants to the principal investigator, who will then take appropriate measures to ensure the safety and wellbeing of the participants.

### Research governance and ethics

#### Trial management

The study will comply with local Research Governance requirements.

#### Ethics

Full ethical approval was obtained from the local Committee (Faculty of Medicine, Universidad de Chile, project number 032-2012). Informed and written consent will be obtained from the participants 18 years of age or older, while for adolescents younger than 18 years of age, their parents’ informed consent will be required, along with the informed assent of the participants.

## Discussion

As far as we know, this is the first randomized controlled trial of a computer-assisted CBT intervention for depressed adolescents in a Latin American country. We believe that the use of the computerized program YPSA-M, coupled with face-to-face CBT, will enhance the therapeutic effects in the intervention participants, compared with the control arm. Past experience has shown that it may be difficult to recruit and sufficiently motivate adolescents to attend psychosocial interventions, given their school schedules and the social stigma associated with mental health treatment [15]. Nevertheless, we hope that the use of the computerized program will be appealing, and that we will receive support from the centers, schools, and parents for successful implementation of the program. It is important to highlight that although the AUGE Clinical Guidelines for Depression recommend the use of psychosocial interventions to treat depression, there is currently no evidence-based intervention available for this age group in Chile. Our intervention could provide an effective psychosocial tool, and primary care psychologists could easily be trained to apply the program.

### Trial status

The study started recruiting patients in July 2013.

## Authors’ information

VM is the principal investigator of this study. She is a child and adolescent psychiatrist, has a PhD in Psychotherapy, and is Assistant Professor of the Faculty of Medicine of Universidad de Chile and a researcher of the Millennium Nucleus Psychological Intervention and Change in Depression.

## Abbreviations

AUGE: Regime of Explicit Health Guarantees
BDI: Beck Depression Inventory
CBT: cognitive-behavioral therapy
CONSORT: Consolidated Standards of Reporting Trials: HRQoL, health-related quality of life
K-SADS-PL: Kiddie-SADS-Present and Lifetime Version
YPSA-M: Yo Pienso, Siento y Actúo Mejor (‘I think, feel, and behave better’).
